# Development of a Competitive Nutrient-Based T-Cell Immunotherapy Designed to Block the Adaptive Warburg Effect in Acute Myeloid Leukemia

**DOI:** 10.3390/biomedicines12102250

**Published:** 2024-10-03

**Authors:** Huynh Cao, Jeffrey Xiao, David J. Baylink, Vinh Nguyen, Nathan Shim, Jae Lee, Dave J. R. Mallari, Samiksha Wasnik, Saied Mirshahidi, Chien-Shing Chen, Hisham Abdel-Azim, Mark E. Reeves, Yi Xu

**Affiliations:** 1 Division of Hematology and Oncology, Department of Medicine, School of Medicine, Loma Linda University, Loma Linda, CA 92354, USA; 2 Cancer Center, Loma Linda University, Loma Linda, CA 92354, USA; 3 Division of Regenerative Medicine, Department of Medicine, School of Medicine, Loma Linda University, Loma Linda, CA 92354, USA; 4 Biospecimen Laboratory, Department of Medicine and Basic Sciences, School of Medicine, Loma Linda University, Loma Linda, CA 92354, USA; 5 Division of Transplant and Cell Therapy, Loma Linda University Cancer Center, Loma Linda, CA 92354, USA; 6 Division of Hematology and Oncology, Department of Pediatrics, Loma Linda University, Loma Linda, CA 92354, USA

**Keywords:** AML, T cells, immunotherapy, CAR T, nutrient, glucose, GLUT1, TFAM, Warburg effect, mitochondrion

## Abstract

**Background:** T-cell-based adoptive cell therapies have emerged at the forefront of cancer immunotherapies; however, failed long-term survival and inevitable exhaustion of transplanted T lymphocytes in vivo limits clinical efficacy. Leukemia blasts possess enhanced glycolysis (Warburg effect), exploiting their microenvironment to deprive nutrients (e.g., glucose) from T cells, leading to T-cell dysfunction and leukemia progression. **Methods:** Thus, we explored whether genetic reprogramming of T-cell metabolism could improve their survival and empower T cells with a competitive glucose-uptake advantage against blasts and inhibit their uncontrolled proliferation. **Results:** Here, we discovered that high-glucose concentration reduced the T-cell expression of glucose transporter GLUT1 (*SLC2A1*) and *TFAM* (mitochondrion transcription factor A), an essential transcriptional regulator of mitochondrial biogenesis, leading to their impaired expansion ex vivo. To overcome the glucose-induced genetic deficiency in metabolism, we engineered T cells with lentiviral overexpression of *SLC2A1* and/or *TFAM* transgene. Multi-omics analyses revealed that metabolic reprogramming promoted T-cell proliferation by increasing IL-2 release and reducing exhaustion. Moreover, the engineered T cells competitively deprived glucose from allogenic blasts and lessened leukemia burden in vitro. **Conclusions:** Our findings propose a novel T-cell immunotherapy that utilizes a dual strategy of starving blasts and cytotoxicity for preventing uncontrolled leukemia proliferation.

## 1. Introduction

Acute myeloid leukemia (AML) is a heterogeneous and severe hematological malignancy [1,2]. Despite advancements in AML treatment, approximately 50% of patients relapse following the induction chemotherapy, resulting in a dismal 5-year survival rate of 30.5% [3]. Consequently, there is an unmet need to develop more effective and less toxic treatments to cure AML and significantly improve patient prognosis [4].

T-cell-based adoptive cell therapies have emerged as a potential treatment option for AML due to the ability to manufacture large amounts of T cells ex vivo and T-cell specificity in attacking leukemic blasts in vivo [5,6,7]. Despite the initial success of adoptive T-cell therapies, there are several recurrent findings hindering their optimal anti-leukemic action to prevent disease relapse in AML: (1) a lack of consistent tumor-specific antigens due to the heterogeneous subpopulations of blasts, (2) a hostile leukemia microenvironment releasing known and unknown inhibitory signals that suppress T-cell functions by causing their exhaustion and senescence, (3) failed long-term survival of transplanted T cells in vivo [8,9,10]. Currently, many approaches have been developed to enhance chimeric antigen receptor (CAR) T-cell therapies with antigen specificities [11,12]; however, the field lacks an effective strategy for increasing the sustainable retention of engrafted T cells in the leukemia microenvironment, which is believed to be fundamental for the success of T-cell therapies for both cancers and non-malignant disorders [13,14,15].

As central components of the microenvironment, adequate nutrients are essential for cancer patients to retain their functional T-cell immunity and aid their healthy recovery [16,17]. T-cell activation/proliferation and effector functions require an abundance of nutrients like glucose [18], which is often inadequate in the AML microenvironment where disease activity is high [19,20]. AML blasts are known to preferentially uptake glucose to produce lactate for energy despite an oxygen abundance, a metabolic phenomenon described as the “Warburg effect” [21]. The consequent depletion of glucose by the rapidly proliferating blasts dampens T-cell activation and induces T-cell exhaustion, leading to leukemia progression [20,22]. In contrast to the corresponding increased gene expression of glucose transporter 1 (GLUT1) in cancer cells, T cells exhibit a downregulation in GLUT1 expression, which constitutes an inhibitory mechanism by cancer cells to elude the T-cell therapies through bottle-necking T cells’ access to necessary glucose [23,24]. As such, targeting tumor metabolism [25,26] including diet-based therapies have been explored to starve the cancer cells by targeting the Warburg effect and reducing their ATP sources to restrict cancer growth [27].

In addition to the microenvironment, another important factor responsible for T-cell function and fate is the functionality of mitochondria [28,29]. In T cells, impaired ATP synthesis and increased mitochondrial reactive oxygen species (ROS) are typical mitochondrial functional defects, leading to T-cell exhaustion and dysfunction [30]. Recently, the deficiency of TFAM, a transcriptional regulator of mitochondrial transcription, replication, and packaging [31,32], was found to be correlated with T-cell senescence and dysfunction of consequential importance [33,34]. This impaired mitochondrial biogenesis forced T cells to reprogram their metabolism towards glycolysis [33,34], demanding T cells to compete against blasts for glucose uptake.

In this study, we explored whether bioengineering T cells to enhance glycolytic metabolism and mitochondrial biogenesis could empower the anti-leukemia function of T cells through a novel nutrient-based strategy. First, we investigated the metabolic action of high glucose on T cells. Then, we genetically engineered a human T-cell line to overcome the inhibitory effect of glucose in reducing the expressions of GLUT1 and TFAM, thereby enhancing their glucose-uptake capability and mitochondrial biogenesis. Finally, we evaluated the extent of the glucose uptake advantage of the engineered T cells (along with the corresponding potential cytotoxicity) in the context of allogenic AML blasts.

## 2. Materials and Methods

The list of reagents including manufacturers and catalogs of antibodies, kits, and qPCR primers is found in the supplementary data (Supplementary Tables S1 and S2). Replicates (*n* = 3) were performed for all experiments.

### 2.1. Human Samples

AML peripheral blood (PB) samples (Supplementary Table S3) were obtained from the Loma Linda University Cancer Center Biospecimen Laboratory (LLUCCBL) and the City of Hope National Medical Center (COHNMC). All donor patients signed an informed consent form. Sample acquisition was approved by the Institutional Review Boards at LLUMC and COHNMC in accordance with an assurance filed with and approved by the Department of Health and Human Services, and it met all requirements of the Declaration of Helsinki Ethic Committee Name: Loma Linda University Health, Approval Code: IRB #58238, Approval Date: 11 October 2023.

### 2.2. Isolation and Ex-Vivo Expansion of T Cells from Primary PB Samples

The detailed protocol of isolation and expansion of primary T cells has been previously reported [7]. Briefly, CD3+ T cells from PB specimens were separated by using CD3 microbeads (Miltenyi Biotech, Bergisch Gladbach, Germany) and a MiniMACS™ Separator with an MS Column according to the manufacturer’s protocol. CD3+ T cells were cultured at 37 °C and 5% CO_2_ in RPMI 1640 culture medium containing 10% fetal bovine serum (FBS, HyClone, Thermo Fisher Scientific, Waltham, MA, USA) with penicillin/streptomycin (100 µg/mL), IL-2 (1000 U/mL, Peprotech, Thermo Fisher Scientific, Waltham, MA, USA), and Dynabeads^®^ Human T-Activator CD3/CD28 (Gibco^TM^, Thermo Fisher Scientific, Waltham, MA, USA) without feeder cells. At around 10–14 days, expanded T cells (5 × 10^6^ cells) were treated with different doses of glucose before further analyses.

### 2.3. Measurement of the Size of T-Cell Clusters

The size (diameter) of T-cell clusters (spheres) was determined by observation under an inverted microscope. Briefly, cell cultures were imaged using an inverted fluorescence microscope (Olympus IX71) under 20x and 40x objectives. Cell clusters with irregular shapes (multiple cluster congregates) were excluded. Then, the diameter of round clusters was measured, and the mean values were calculated in 5 samples in at least 10 independent cultures.

### 2.4. Cell Culture of MV4-11, Jurkat and New Transgenic Jurkat Cell Lines

MV4-11 is a human-derived AML blast cell line with an FLT3 mutation (ATCC CRL-9591). Jurkat is a human-derived T-lymphoblast cell line (ATCC TIB-152^TM^). All cell lines were cultured in RPMI-1640 medium (HyClone, Thermo Fisher Scientific, Waltham, MA, USA) supplemented with 10% heat-inactivated fetal bovine serum (HyClone) and 100 U/mL penicillin/streptomycin. For high-glucose treatment, the culture media were adjusted to the amount of 25 mM, 50 mM, and 100 mM glucose. Cells were grown at 37 °C in a humidified atmosphere containing 5% CO_2_.

### 2.5. Glucose Treatment of Jurkat In Vitro

(a) Short period of glucose treatment: Jurkat cells (1 × 10^6^ cells) were treated with different doses of glucose for 48 h (48 h) before further analyses.

(b) Extended period of glucose treatment: Jurkat cells were treated with different doses of glucose for 4 weeks before further analyses. The cell culture medium was changed every 2–3 days with glucose.

### 2.6. Preparation of SLC2A1 (GLUT1) Lentivirus, TFAM Lentivirus and Generation of GLUT1-Jurkat, TFAM-Jurkat, GLUT1/TFAM-Jurkat, and GFP-Jurkat Cell Lines In Vitro

The lentiviral transfer plasmids contain a full-length open reading frame of human *SLC2A1 (GLUT1)* (GeneCopoeia catalog: EX-C0124-Lv225, Rockville, MD, USA),) and *TFAM* (GeneCopoeia catalog: EX-F0774-Lv225). This plasmid expresses *GLUT1* or *TFAM* and *GFP* or *Cherry* controlled by the EF1 and IRES2 promoters, respectively. A *GFP* empty vector (GeneCopoeia catalog: EX-NEG-Lv225) was used as the vector control.

Lentiviruses were prepared as previously described [35]. Briefly, HEK-293T cells were cultured in complete Dulbecco’s Modified Eagle Medium (DMEM, Gibco, Waltham, MA, USA) containing 10% FBS and 100 U/mL penicillin/streptomycin. When the cells were 70–80% confluent, the culture media were replenished, and a transfection solution containing an envelope, packaging, and transfer plasmid (*GLUT1* or *TFAM* or *GFP*) was added dropwise to the cells. After the cells were cultured at 37 °C and 5% CO_2_ for 48 h, supernatants were collected, filtered through a 0.45 µm filter, and centrifuged at 4800× *g* at 4 °C for 24 h. The typical titer of the virus was 10^8^–10^9^ transducing units/mL. The Jurkat cells were transduced with lentivirus at a multiplicity of infection of 5. Twenty-four hours later, the virus was removed, and the culture media were replenished. The cells were cultured for 24 h and examined for transduction efficiency via fluorescence microscopy and flow cytometry. GLUT1-Jurkat, TFAM-Jurkat, and GLUT1/TFAM-Jurkat cell lines were purified by Fluorescence-Activated Cell Sorting (FACS; Research Core Facility, School of Medicine, University of California, Riverside, CA, USA). We did not observe significant phenotypic changes in sorted Jurkat cell lines before and after FACS sorting, which is consistent with previous reports in terms of unaltered proliferation, etc. [36,37].

### 2.7. Glucose Uptake and Cytotoxicity Assays

Glucose (2-NBDG: *D-glucose, 2-[N-(7-nitrobenz-2-oxa-1,3-diazol-4-yl) amino]-2-deoxy-D-glucose*) uptake assays were performed by the co-culture of Jurkat cell lines with MV4-11 blasts in 24-well plates with transwell inserts (Corning 3422). The ratio of Jurkat cells (outside the transwell insert) to MV4-11 (inside the transwell insert) was 1:1 and 1:5. After 48 h co-culture, 2-NBDG (50 μM, MedChemExpress, Monmouth Junction, NJ, USA) was added to the medium to treat cells for 2 h. MV4-11 cells from the transwell insert were collected either for flow cytometry measurement of 2-NBDG uptake and cell death (Annexin-V/PI) assay or for RNA isolation and qPCR analyses.

Cytotoxicity assays were performed by co-culturing GLUT1-engineered primary T cells (same engineering procedure as that of GLUT1-Jurkat) with AML patient blasts (isolated by CD33-microbeads pull-down) in 48-well plates. The ratio of allogenic T cells to AML blasts ranged from 1:1 to 3:1. After overnight incubation, cells were collected, stained, and processed for an FACS assay of biomarkers including Viability Dye eFluor™ 780 (eBioscience™, Thermo Fisher Scientific, Waltham, MA, USA), CD3 and CD33 (Biolegend, San Diego, CA, USA) according to manufacturers’ protocols.

### 2.8. Flow Cytometry (FC)

Experimental groups of T cells were harvested and examined for the expression of cell surface biomarkers and intracellular proteins by multichromatic flow cytometry, as previously described [38]. About 0.5~1 × 10^6^ cells were resuspended in 100 µL FC buffer (PBS containing 1% FBS and 0.05% sodium azide) and stained with various fluorescent-conjugated antibodies specific for the desired cell surface proteins at 4 °C for 30 min. Isotype controls and fluorescence minus one (FMO) control were performed to exclude non-specific binding and autofluorescence. The surface-stained cells were then fixed and permeabilized using the appropriate reagents (BD Pharmingen Cytofix/Cytoperm buffer) and further stained with appropriate fluorescent-conjugated antibodies specific for the desired intracellular proteins such as GLUT1 at 4 °C for 2 h in the permeabilizing buffer (BD Perm/Wash buffer). Concentrations of the antibodies were used per manufacturers’ recommendations (Supplementary Table S1). Finally, the stained cells were washed twice in permeabilizing buffer and twice in FC buffer before analysis on the BD FACSAria II. Data were analyzed using the FlowJo software (v10.6, Tree Star Inc., Ashland, OR, USA).

### 2.9. RNA Isolation and qPCR Analysis

Cells were collected for RNA isolation and qPCR analysis as previously described [39]. Total RNA was isolated using the RNeasy Mini Kit (Qiagen, Germantown, MD, USA) according to the manufacturer’s instructions. First-strand cDNA was synthesized using the SuperScript III Reverse Transcriptase (Invitrogen; Life Technologies, Waltham, MA, USA). The qPCR was performed and analyzed in Applied Biosystems 7900HT Real-Time PCR machine. Sequences of primers used in this study are available in Supplementary Table S2. The PCR conditions were 10 min at 95 °C followed by 40 cycles of 10 sec at 95 °C and 15 sec at 60 °C. The relative expression level of a gene was determined using the ΔΔCt method and normalized to *β-actin*.

### 2.10. Proteomics Analysis

Cell-free supernatants were collected for the human cytokine profile assay as previously described [40]. The Human XL Cytokine Array Kit (Catalog # ARY022B, R&D, Minneapolis, MN, USA) was used according to the manufacturer’s procedures and blot images were acquired from Azure 300 Imager (Azure Biosystems, Dublin, CA, USA). This kit has 105 different cytokine antibodies and detailed names with their corresponding coordinates are listed on pages 15–17 of the appendix section of the manufacturer’s product datasheet [41]. Blue arrows indicated the reference spots.

### 2.11. Imaging Acquisition

Phase-bright images were captured using an Olympus 1X71 inverted microscope and were processed using an Olympus cellSens Dimension 1.15 Imaging Software (Tokyo, Japan).

### 2.12. Statistical Analysis

Statistical analyses were performed with GraphPad software (Prism 5.02, San Diego, CA, USA). The quantitative analyses were analyzed using a one-tailed or two-tailed, unpaired *t*-test for comparison of two groups, or a one-way or two-way ANOVA test for comparison of three or more groups. All values were presented as mean ± SEM. The results were considered statistically significant when the *p*-value was <0.05.

## 3. Results

### 3.1. The Supplementation of High Glucose Suppressed T-Cell Proliferation Ex Vivo

Previously, high glucose was reported to promote the pathogenesis of autoimmunity disorders through inducing the mitochondrial defects of T cells [42]. Also, hyperglycemia/diabetes mellitus has been associated with an increased risk of up to 20% for leukemia [43,44] and a significant increase in the odds of mortality in AML patients [45]. Therefore, to utilize glucose uptake as a therapeutic strategy, which has a potential risk of high-glucose concentration inside T cells, we need to first understand how high glucose can affect T cells in vitro so that we can look for solutions to overcome the weakness. First, we isolated and expanded T cells from peripheral blood (PB) samples of AML patients (AML-T, Supplementary Table S3) ex vivo based on our established protocol [7]. Then, we treated T cells with different doses of glucose for 48 h. We found that the size of T-cell clusters significantly decreased by 1.6- and 3-fold in AML-T cells of patients when treated with higher glucose doses (50 mM and 100 mM), respectively (Supplementary Figure S1A,C, *N* = 3). All patient specimen-derived T cells displayed the same phenomenon of reduced T-cell cluster sizes after high-glucose treatment (Supplementary Table S3, *N* = 5). Next, we performed flow cytometry (FC) analyses and found a significantly decreased percentage of Ki67+CD3+ proliferating T cells in experimental groups of AML-T cells pretreated with 50 mM or 100 mM glucose when compared to no treatment (NO TX) by 4.9% and 16.79%, respectively (Supplementary Figure S1B), suggesting that high glucose could impair T-cell proliferation.

### 3.2. High-Glucose Concentration Significantly Reduced GLUT1 and TFAM Expression in T Cells

**Figure 1:**
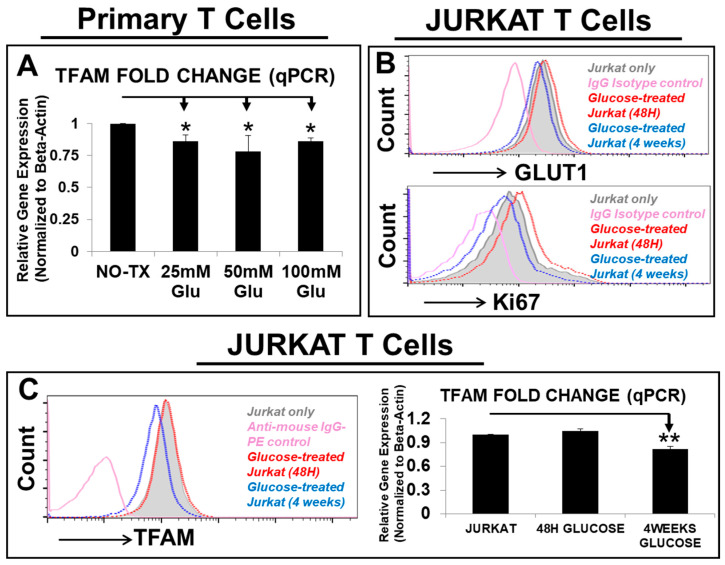
High glucose significantly reduced the expression of GLUT1 and TFAM in T cells. (**A**) Gene expression of *TFAM*, a mitochondrial transcription factor was analyzed by qPCR. Data of mRNA expressions show the fold change (normalized to *β-actin*) of the gene encoding TFAM in T cells from AML patient samples, which were pretreated with different doses of glucose; (**B**) representative FC histograms show the expression of GLUT1 and Ki67 in Jurkat cells which were pretreated with 25 mM glucose for 48 h (red line plot) or persistent supplementation of 25 mM glucose for 4 weeks in vitro (blue line plot); the filled grey line and pink line plots represent Jurkat without treatment and IgG-fluorescent control; (**C**) representative FC histograms show the expression of TFAM in Jurkat cells which were pretreated with 25 mM glucose for 48 h (red line plot) or persistent supplementation of 25 mM glucose for 4 weeks in vitro (blue line plot); the filled grey line and pink line plots represent Jurkat without treatment and IgG-fluorescent control; Right panel: qPCR data of mRNA expressions show the fold change (normalized to *β-actin*) of the gene encoding *TFAM* in Jurkat which were pretreated with or without different time points of 25 mM glucose; where applicable, data are means ± SEM. * *p* < 0.05, ** *p* < 0.01, *n* = 3.

TFAM (mitochondrion transcription factor A) is known to be essential for transcription and replication of mitochondrial genes which encode 13 protein subunits of respiratory chain essential for ATP production [46]. To investigate glucose’s effect on mitochondrial genes, we performed qPCR and found that *TFAM* was consistently decreased in gene expression by high glucose in different experimental groups of AML-T cells (*p* < 0.05) (Figure 1A, *N* = 3). To confirm this phenomenon, we studied the effects of glucose treatment (25 mM) on Jurkat, a human T-lymphocyte cell line [47], over a short-term (48 h) and long-term (4 weeks) time span. In the 48 h experimental group, 25 mM glucose treatment increased GLUT1 (glucose transporter 1) expression when compared to the NO TX Jurkat (Figure 1B, upper plot), which was consistent with increased Ki67+ proliferation (Figure 1B, lower plot). However, in the 4-week experimental group, 25 mM glucose treatment reduced both GLUT1 expression and Ki67+ proliferation when compared to the NO TX Jurkat (Figure 1B). In contrast to the minor changes in gene and protein expressions of TFAM in the 48 h glucose-treated Jurkat, there was a significant reduction in gene and protein expressions of TFAM in the 4-week glucose-treated Jurkat (*p* < 0.05) (Figure 1C). These data confirmed that prolonged exposure to high glucose could reduce the expression of both GLUT1 and TFAM in T cells.

### 3.3. Generation of Metabolism-Enhanced T-Cell Lines Overexpressing GLUT1 and/or TFAM

**Figure 2:**
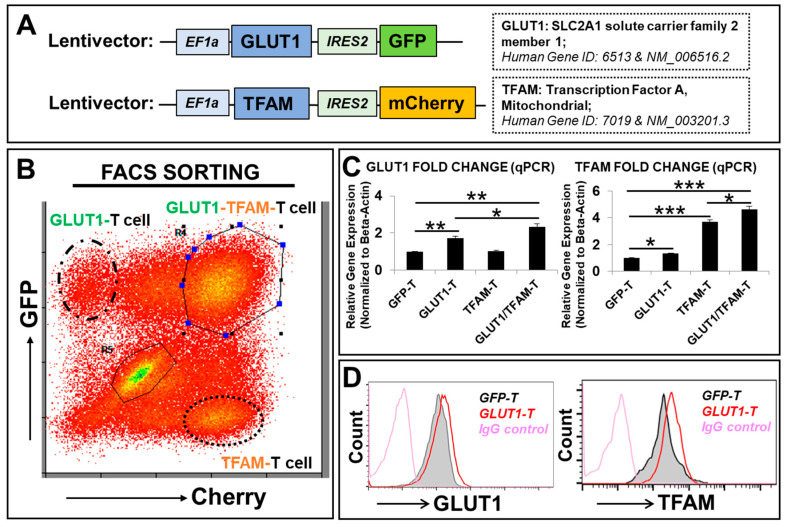
Generation of transgenic T-cell lines overexpressing transgenes of *SLC2A1 (GLUT1)* and/or *TFAM* in vitro. (**A**) Schematic diagram of lentiviral expression construct containing human *GLUT1* or *TFAM* open-reading frames and *GFP* or *Cherry* reporters, with promoters EF1a and IRES2, respectively; (**B**) a representative FC plot shows GFP and/or Cherry expression in GLUT1-T, TFAM-T, and GLUT1/TFAM-T cells (FACS-sorted, see Materials and Methods); (**C**) overexpressed gene expression of *GLUT1* and *TFAM* was analyzed by qPCR. Data of mRNA expressions show the fold change (normalized to *β-actin*) of genes encoding GLUT1 and TFAM in new T-cell lines; (**D**) representative FC histograms show GLUT1 and TFAM expression in GLUT1-T (red line plots) versus vector control (GFP-T cells, the filled grey line) and IgG-fluorescent control (pink line plots); Where applicable, data are means ± SEM. * *p* < 0.05, ** *p* < 0.01, *** *p* < 0.005, *n* = 3.

To reverse the glucose-induced deficiency of GLUT1 (*SLC2A1*) and *TFAM*, we utilized the lentiviral system (established in our lab) to overexpress the *GLUT1* and/or *TFAM* transgenes in Jurkats to generate novel transgenic Jurkat T-cell lines in vitro including GLUT1-T cells (or GLUT1-Jurkat), TFAM-T cells (or TFAM-Jurkat), GLUT1/TFAM-T cells (or GLUT1/TFAM-Jurkat), and GFP-alone T cells (or GFP-Jurkat, the vector control) (Figure 2A). Jurkat cells have been used as the reporter system to evaluate the novel chimeric antigen receptors and their cytotoxic effect [48]. All newly generated transgenic T-cell lines were purified by FACS sorting (Figure 2A,B). The overexpression of *GLUT1* and *TFAM* transgenes was assessed by affiliated fluorescent reporters including GFP and mCherry in their original lentiviral constructs (Figure 2B) and further confirmed by qPCR analyses of transcript expressions (Figure 2C). Notably, *GLUT1* overexpression increased *TFAM* gene expression (Figure 2C, right panel), while *TFAM* overexpression did not exhibit a reciprocal increase in *GLUT1* gene expression (Figure 2C, left panel). Furthermore, there was an additive effect in *GLUT1* and *TFAM* gene expressions in the combined GLUT1/TFAM-T group (2.3-fold GLUT1 increase, 4.6-fold *TFAM* increase relative to *GFP*-T) when compared to the GLUT1-T group (1.7-fold *GLUT1* increase, 1.3-fold *TFAM* increase) or the TFAM-T group (no *GLUT1* increase, 3.6-fold *TFAM* increase) (Figure 2C). Previous studies have shown that *TFAM* overexpression could indirectly increase the expression of *GLUT4* in the skeletal muscle [49]. Although TFAM alone did not induce *GLUT1* expression, *GLUT1* and *TFAM* overexpression could lead to an increase in their respective downstream molecules that might combine to additively increase the gene expression of *GLUT1* in GLUT1/TFAM-T cells. In addition to increased GLUT1 protein expression (Figure 2D, left plot), *GLUT1* overexpression increased the protein expression of TFAM (Figure 2D, right plot), which is consistent with increased *TFAM* gene expression in GLUT1-T (Figure 2C, right panel).

**Figure 3:**
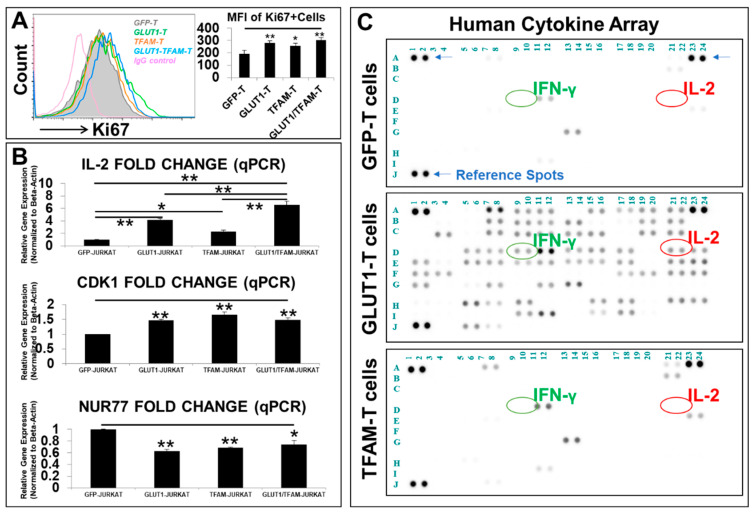
Phenotypic characterizations of metabolism-engineered T-cell lines in vitro. (**A**) A representative FC histogram (left panel) shows the expression of Ki67 in new transgenic T-cell lines, including GLUT1-T (green line plot), TFAM-T (orange line plot), GLUT1/TFAM-T (blue line plot), versus GFP-T cells (the filled grey line), and IgG-fluorescent control (pink line plot); cumulative data (right panel) of the mean fluorescence intensity (MFI) levels of Ki67 expression in the new T-cell lines; (**B**) qPCR analysis of the gene expression of different cytokines and biomarkers for cell divisions, inhibitory signals, and exhaustion. Data of mRNA expressions show the fold change (normalized to *β-actin*) of genes encoding *IL-2, CDK1*, and *NUR77* in new T-cell lines; (**C**) image of blot films, with reference spots (blue arrows), interferon-gamma (IFN-γ, IFNG) (green circle), and interleukin-2 (IL-2) (red circle), developed for proteomic analyses of cell-free supernatants from new T-cell lines, including GLUT1-T and TFAM-T versus GFP-T cells; where applicable, data are means ± SEM. * *p* < 0.05, ** *p* < 0.01, *n* = 3.

Next, we performed FC analysis to evaluate the effects of *GLUT1* and *TFAM* overexpression on T-cell proliferation. The FC histogram (Figure 3A, left panel) and mean fluorescence intensity (MFI) levels of Ki67 (Figure 3A, right panel) demonstrated that increased *GLUT1* and *TFAM* was positively correlated with increased cell proliferation of GLUT1-T, TFAM-T, and GLUT1/TFAM-T when compared to GFP-T control, suggesting that metabolic enhancement of both glycolytic and mitochondrial pathways can promote T cell proliferation.

To elucidate the mechanism underlying the enhanced proliferation of these transgenic T cells, we conducted additional qPCR analysis. All GLUT1-T, TFAM-T, and GLUT1/TFAM-T cells demonstrated significantly increased gene expression by 4.1-fold, 2.2-fold, and 6.5-fold, respectively (Figure 3B), of *IL-2*, a key cytokine known to promote T cell proliferation by preventing their exhaustion and overcoming tumor immunosuppressors [50,51]. There was an additive effect in increasing *IL-2* expression in GLUT1/TFAM-T when compared to GLUT1-T- or TFAM-T-only cell lines (Figure 3B). Furthermore, all GLUT1-T-, TFAM-T-, and GLUT1/TFAM-T-cell lines demonstrated significantly increased gene expression of *cyclin-dependent kinase 1 (CDK1)*, an essential central regulator that drives cell proliferation [52] (Figure 3B). To elucidate the mechanism by which *GLUT1* and/or *TFAM* overexpression reverses the T-cell exhaustion/senescence, we conducted qPCR screens on exhaustion genes and inhibitory signaling pathways. We found that engineered T cells with *GLUT1*, *TFAM*, and *GLUT1/TFAM* overexpression had significantly decreased gene expression of *nuclear receptor 4A1* (*Nur77*, Figure 3B), a key exhaustion biomarker, found to suppress T-cell activation and proliferation [53,54].

Finally, to investigate cytokine release in engineered T cells with GLUT1 or TFAM overexpression, we performed comprehensive proteomics of human cytokines secreted in the cell-free supernatants from these new T-cell lines (Figure 3C). Both GFP-T and TFAM-T cells released limited cytokine profiles in their supernatant (Figure 3C, top and lower plots). Interestingly, GLUT1-T cells released many different cytokines including IFN-γ (D9, D10 coordinates indicated by green circles in Figure 3C), which is known to be essential for the development of cytotoxic CD8 T cells [55,56] and to markedly enhance anti-tumor activities of CAR T therapy in vivo [57]. Furthermore, *GLUT1* overexpression significantly increased the production and release of IL-2 (Figure 3C, middle plot), which can determine the T-cell fate within a framework of other signal transduction networks [58]. This increased the release of IL-2 also confirmed the GLUT1-upregulated gene expression of *IL-2* (Figure 3B). Notably, IL-2 and IFN-γ were reported to synergistically induce the proliferation and differentiation of cytotoxic T cells [59]. A full list of 41 additional cytokines (associated with T-cell metabolism) increased by the GLUT1-T cells compared to the control cells, their corresponding coordinates from Figure 3C, and their relevant functions are illustrated in Supplementary Table S4, indicating the potential mechanism of how *GLUT1* overexpression can improve T-cells’ metabolic fitness through different cytokines.

In summary, our findings suggest that empowering T cells with metabolic enhancement with both glycolytic and mitochondrial pathways could promote T-cell proliferation through increased production and release of cytokines like IL-2, enhanced cell cycle progression and inhibition of the Nur77-mediated exhaustion pathway in vitro.

### 3.4. GLUT1-T and GLUT1/TFAM-T Cells Performed Anti-Leukemia Effects through Advanced Glucose-Uptake Capability In Vitro

**Figure 4:**
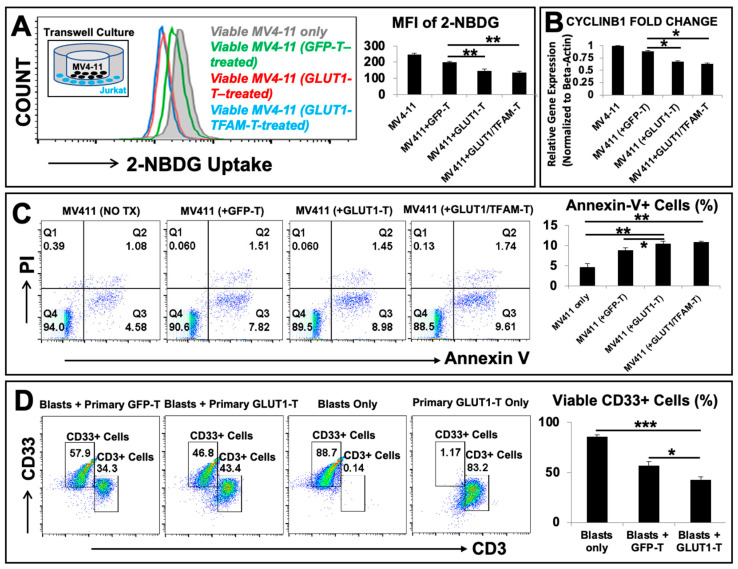
GLUT1-T or GLUT1/TFAM-T cells performed anti-leukemia effects through advanced glucose-uptake capability in vitro. (**A**) Left panel: Representative FC histograms show the 2-NBDG uptake in viable MV4-11 co-cultured with GLUT1-T (red line plots) and GLUT1/TFAM-T (blue line plots) versus vector control (GFP-T cells, green line) and viable MV4-11 alone (the filled grey line plots); a visual diagram of the transwell culture (inset) with MV4-11 cells (black) and engineered Jurkat T cells (blue); Right panel: mean fluorescence intensity (MFI) levels of 2-NBDG uptake in different experimental groups; (**B**) gene expression of CYCLIN B1 (*CCNB1* gene), a biomarker for mitosis in the cell cycle was analyzed by qPCR. Data of mRNA expressions show the fold change (normalized to *β-actin*) of CYCLIN B1 gene in MV4-11 alone or MV4-11 co-cultured with different engineered T-cell lines in transwell; (**C**) representative FC plots show the expression of an Annexin V/PI apoptosis and necrosis assay in transwell co-cultures, including GLUT1-T co-culturing with MV4-11 versus GFP-T co-culturing with MV4-11 and MV4-11 only in vitro; Right panel: cumulative FC percentage data of Annexin V+ cells in the different experimental groups; (**D**) representative FC plots show the expression of CD33, a biomarker for primary AML blasts and CD3 in different cytotoxic co-cultures, including GLUT1-engineered primary T cells (GLUT1-T) co-culturing with primary CD33+ blasts versus GFP-T cells (vector control) co-culturing with CD33+ blasts, CD33+ blasts only and GLUT1-T only in vitro; Right panel: cumulative FC percentage data of viable CD33+ cells in different experimental groups; where applicable, data are means ± SEM. * *p* < 0.05, ** *p*< 0.01, *** *p* < 0.005, *n* = 3.

To evaluate the anti-leukemia effects of metabolism-enhanced T cells, we performed a proof-of-principle exploration by directly co-culturing engineered T cells and either allogenic MV4-11, an AML blast cell line, or primary AML patient derived-CD33+ blasts in vitro. CD33 is a biomarker for AML blasts. First, we performed transwell co-cultures to determine whether advanced competency for the glucose uptake in metabolism-enhanced T cells will be a causative factor for the reduction of viable MV4-11 blasts (Figure 4A–C). A diagram was presented to illustrate the transwell setup of MV4-11 (black) and engineered T cells (blue) (Figure 4A, inset). First, we combined transwell co-cultures with a glucose-uptake assay (2-NBDG, a fluorescent glucose analog) (see detailed procedures in the section of methods). In contrast to the normal 2-NBDG uptake in the MV4-11 control group (without engineered T cells) and the minor reduction of 2-NBDG uptake in MV4-11 co-cultured with GFP-T, our FC histogram data (Figure 4A, left panel) and MFI data (Figure 4A, right panel) revealed that there was a significant reduction of 2-NBDG uptake in MV4-11 co-cultured with GLUT1-T and GLUT1/TFAM-T cells in vitro. Then, we analyzed the genetic changes of cell division through qPCR with the *CYCLIN B1* (*CCNB1* gene), a biomarker essential for the initiation of mitosis in tumors [60]. The gene expression of *CYCLIN B1* was significantly reduced in MV4-11 co-cultured with GLUT1-T (~20% decrease) when compared to MV4-11 co-cultured with GFP-T cells (Figure 4B). Furthermore, a cell death kit (MBL #4700) combining both necrosis (Propidium Iodide, PI) and apoptosis (Annexin V) assays was applied to detect the early stage of apoptotic MV4-11 cells in transwell co-culture (Figure 4C). We found that early apoptotic MV4-11 cells increased from 4.58% in the control to 7.82% with GFP-T, 8.98% with GLUT1-T, and 9.61% with GLUT1/TFAM -T (Figure 4C). Finally, we performed cytotoxic co-cultures of engineered primary T cells and AML patient-derived CD33+ blasts. Primary viable CD33+ blasts decreased from 88.7% in the blasts-only group to 57.9% in the GFP-T + blasts group and then to 46.8% in the GLUT1-T + blasts group (Figure 4D). Collectively, our preliminary data suggest that GLUT1-T or GLUT1/TFAM-T cells can competitively reduce glucose uptake in blasts, leading to a significant leukemic reduction in vitro.

## 4. Discussion

**Figure 5:**
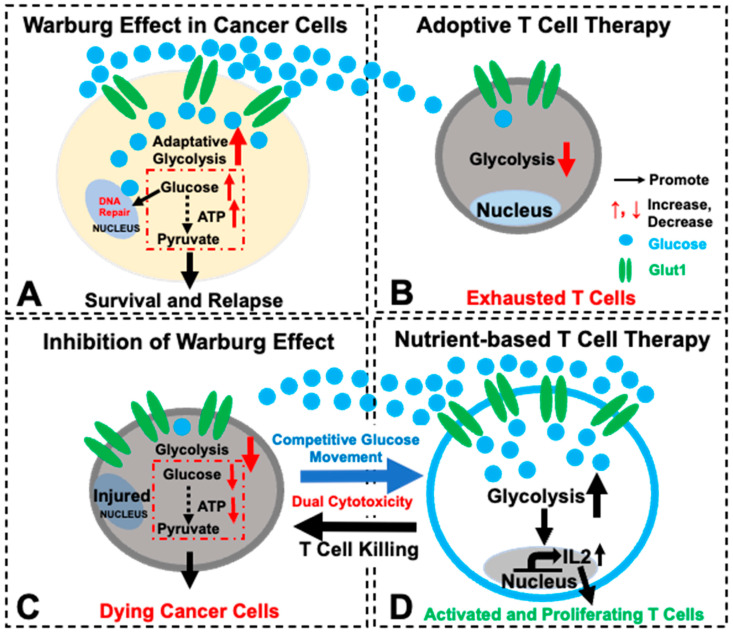
A schematic diagram illustrating the strategy of developing a nutrient-competency-based and metabolism-enhanced T-cell therapy with dual cytotoxicity for AML. (**A**) To escape current therapies, blasts utilize the Warburg effect (increased glycolysis) to support their uncontrolled proliferation and initiate AML relapse. (**B**) In current adoptive T-cell therapies, T cells might not be able to compete in glucose uptake against blasts, which are known to suppress T cells by inhibitory signals (e.g., PD-L1) and to deprive glucose from T cells. (**C**) When the Warburg effect is inhibited in blasts, cancer cells cannot survive as well due to the decreased glycolysis and ATP production. (**D**) Consequently, to improve T-cell therapy for AML, we metabolically reprogrammed T cells by the overexpression of *GLUT1* and *TFAM* transgenes. *GLUT1* overexpression significantly enhanced T-cells’ competitive glucose-uptake capability against blasts and deprived glucose from blasts to reduce the Warburg effect, potentially leading to the increased blast death. Moreover, *GLUT1* and/or *TFAM* overexpression can enhance the production and release of IL-2 to promote T-cell survival and proliferation while reducing *NUR77*, a transcription initiator of T-cell exhaustion. In summary, our preliminary data suggest that genetically reprogramming T cells with enhanced *GLUT1*/or *TFAM* might be a novel approach to develop a durable and effective T-cell therapy to treat AML.

CAR T-cell therapy, a living drug, has displayed significant therapeutic potential for cancer patients and non-malignant disorders [61,62]. However, since AML is a heterogeneous blood cancer with dynamic etiologies of somatically acquired alterations at the DNA, RNA, epigenetic, and protein levels, only ~30% of patients show a response to CAR T-cell therapies [63]. In addition to off-tumor effects and lack of ideal surface antigens to target, failed long-term survival of transplanted T cells in vivo (T cell exhaustion) has limited the successful application of CAR T-cell therapies for AML [64]. We reasoned that rescuing T-cell exhaustion would be essentially required for the success of adoptive T cell therapies [9]. Therefore, we sought to overcome the negative effects of the leukemia microenvironment which blasts adaptively manage and manipulate to limit the efficacy of these treatments [65]. In this proof-of-concept study designed to improve the T-cell immunotherapy for AML, we discovered two important advances to address T-cell exhaustion and improve adoptive T-cell therapy (Figure 5): (1) reprogramming T-cell metabolism (improving T survival); (2) enhancing T-cell competition for glucose with *GLUT1* overexpression (starving blasts to block their Warburg effect), which will not only reverse the glucose trend to promote T cells but also serve as an alternative backup plan in case of the failure of pre-designed CAR T therapies (refractory subclones with antigen variants) while still utilizing the strategy of metabolically starving blasts to overcome AML relapse and significantly improve AML prognosis.

Hyperglycemia/diabetes mellitus is known to cause mitochondrial dysfunction, including increased reactive oxygen species (ROS) production [66]. Short-term (up to 3 days) high-glucose (25 mM and 50 mM) treatment was found to exacerbate autoimmune diseases through the activated ROS/TGF-β pathway in vitro and in vivo [42]. In addition, hyperglycemia is known to impair T-cell immunity in diabetic patients and to be associated with increased risks of COVID-19 infection [67], leukemia (20% increase) [43,44], and AML mortality [45]. Because our strategy is to enhance T-cell glucose uptake and reverse the glucose trend towards blasts, following with the previous reports, we examined how 25 mM and higher glucose affect T cells in vitro (Figure 1). Meanwhile, the in vitro 4-week treatment period reflects clinical observations of CAR T-cell dynamics, with studies reporting loss of CAR T cells as early as 2 weeks post-infusion [68] and peak expansion of infused CAR T-cells between 1–3 weeks [69]. Thus, durable CAR T-cell therapy remains challenging, with some trials showing 40% of patients losing persistent CAR T-cells after 3 months [70]. In this regard, our innovative approach of enhancing T-cells’ nutrient competency and metabolism through genetic overexpression of GLUT1 and TFAM aims to address the metabolic competition of CAR T cells against blasts in the leukemia-controlled microenvironment.

### 4.1. Reprogramming T Cells with Enhanced Metabolism through GLUT1/TFAM

T-cell activation (either naïve, effector, or memory T cells) and their growth rely on available environmental nutrients and extracellular signals [71,72]. Glucose uptake plays an essential part in generating abundant metabolites and ATP energy to support T-cell activation, signal transduction, and proliferation [73]. Thus, T-cell exhaustion and dysfunction occur when T cells do not adequately uptake glucose due to leukemia-induced suppression of GLUT1 [23]. To overcome and prevent the reduced expression of GLUT1, we utilized a lentiviral system to empower Jurkat T cells with the overexpression of *GLUT1* transgene (Figure 2). Notably, our transgenic GLUT1-T cell was found to significantly increase both gene expression and protein release of IL-2 (Figure 3B,C), a key cytokine for T-cell activation, function, and survival [58], leading to the accelerated T-cell proliferation in vitro. However, it is also important to recognize that an IL-2 increase does not only produce positive benefits since IL-2 combined with a higher glycolytic metabolism may also cause differentiation of T cells toward the T effector and Temra phenotype [74], thereby impacting the persistence of T cells. For the success of T-cell-based immunotherapies, striking a healthy balance between having a cytotoxic T-cell population and a good memory that enables proper persistence in vivo must be addressed. Consequently, there is a potential limitation in interpreting the IL-2 increase because pushing IL-2 in producing T cells has both advantages and disadvantages [75]. While our initial focus was on IL-2 due to those roles, we recognize the importance of evaluating a broader range of cytokines. For example, GLUT1 overexpression also increased production of a multitude of factors including IFN-γ (Figure 3C), some of which could have contributed to the observed increased activation and proliferation of T cells (Supplementary Table S4).

In addition to GLUT1 overexpression (glycolytic pathways), mitochondrial biogenesis and related ATP/metabolite production are also essential for T-cell activation, function, and survival [76]. For example, the deficiency of TFAM, a key nuclear transcription factor of controlling the mitochondrial genome to encode 13 protein subunits for the mitochondrial respiratory chain for ATP production [46], was correlated to the significantly reduced numbers of circulating CD4/CD8 effector T cells [33]. Therefore, we reasoned that enhancing mitochondrial biogenesis of T cells through TFAM overexpression could aid T cells energetically to prevent their exhaustion and maintain their effector function. As a result, overexpressing both GLUT1 and TFAM can overcome the potential negative effects of high glucose on mitochondrial function [77]. Our data demonstrated that overexpression of *TFAM* (as will *GLUT1* overexpression) could promote the gene expression of IL-2 in engineered T cells (Figure 3B), revealing how reprogramming T-cell metabolism can induce greater IL-2 expression. This can be valuable towards finding a novel therapy because a cell-based release of IL-2 is known to enhance long-term efficacy of adoptive T-cell therapy [78,79] while avoiding systemic toxicity [51,80]. Moreover, *TFAM* overexpression was found to reduce the expression of *NUR77* (Figure 3B), a nuclear transcriptional initiator of T-cell exhaustion, supporting previous findings that up-regulated *TFAM* can promote cell survival [81]. *NUR77* expression was investigated because of its vital role as an early indicator of T-cell activation, sensitivity to metabolic perturbations, and regulatory ability of other exhaustion markers like PD-L1 [53,82].

Collectively, our in vitro data of successfully engineering T-cell metabolism suggest the importance of reprogramming T cells through GLUT1/glycolytic pathways supplemented with TFAM/mitochondrial biogenesis to improve T-cell survival and prevent T-cell exhaustion (Figure 5).

### 4.2. Enhancing T-Cell Competition for Glucose as a Novel Strategy of Starving Blasts and Thereby Blocking the Warburg Effect of Blasts (Figure 5)

Uncontrolled proliferating blasts take advantage of their microenvironment by depriving glucose and other nutrients from the surrounding hematopoietic environment including T cells [20]. In particular, AML blasts are known to preferentially uptake glucose for energy production (termed as the “Warburg effect”) [21], an inhibitory mechanism which compromises the adoptive T-cell therapy through bottle-necking T-cells’ access to necessary glucose for activation and function [23,24] (Figure 5A,B).

In this regard, we explored whether genetically engineering T cells with *GLUT1* overexpression could empower T cells with a competitive advantage in glucose uptake against blasts and inhibit their uncontrolled proliferation. Notably, our new transgenic human T-cell lines (either GLUT1-T or GLUT1/TFAM-T) were found to suppress AML blasts’ glucose uptake and thereby result in the lessened leukemia burden in vitro (Figure 4 and Figure 5C,D).

The current study has a few limitations due to our limited primary patient samples and the primarily in vitro data of genetic engineering Jurkat, a human cell line and transgenic Jurkat cells. The Jurkat cell line is an immortalized T-cell line derived from leukemia cells, which may exhibit certain characteristic and functional differences from regular T cells. Thus, future studies should engineer primary T cells from more patients and investigate their functions to confirm our findings. Interestingly, a recent published study successfully applied a major part of our novel idea in vivo by engineering CAR T cells with GLUT1 overexpression, leading to the increased metabolic fitness of primary T cells, reduced T-cell exhaustion, and enhanced anti-tumor efficacy against multiple different cancers in humanized murine models [83]. This new report and our current study not only confirmed each other’s findings through a combined in vitro and in vivo lens but also suggest the importance of investigating this novel glucose-competency-based therapeutic strategy through the murine AML microenvironment in the future to see if our observation is durable in vivo. Also, whether metabolism-enhanced T cells can overcome immunosuppressor ligands such as PD-L1 [84] and their engaged PD-1 signaling pathways [85] in vivo is another important topic to be examined in our future studies.

In the current study, we performed a proof-of-concept study to show an alternative route of combating AML blasts by enhancing the glucose competency of an adoptive cell vehicle like non-specific Jurkat T cells, which were demonstrated to deprive blasts of glucose and potentially increase blast deaths (Figure 4A–C). In this regard, our successful exploration of GLUT1-overexpressed Jurkat (non-specific T cells) in this study suggests the potential feasibility of utilizing alternative non-specific cell vehicles against leukemic blasts like healthy hematopoietic stem cells (HSCs), or HSCs-derived engineered natural killer T cells [86] (NK-T cells) and empowering them with GLUT1-overexpressed gene therapy in adoptive cell transfer to treat AML. If GLUT1-overexpressed HSCs can compete for glucose against blasts while regenerating the healthy blood and immune systems of AML patients, then that could further elevate the likelihood of success for an already established and effective leukemic treatment option in the allogeneic HSC transplantation. Additionally, multiple studies have suggested that NK cells actively compete against tumor cells for nutrients in the immunosuppressive tumor microenvironment [87] and that CAR engineering of NK cells can enhance metabolic fitness against highly metabolically active tumors [88]. Thus, the application of our strategy to other immune cells could provide other novel methodologies to improve the current therapies for leukemia patients.

## 5. Conclusions

Our proposal of reprogramming T cells metabolically (improving T-cell’s long-term survival in the leukemia microenvironment) and enhancing T-cell competition for glucose (starving blasts to inhibit their blatant proliferation) could be a novel nutrient (glucose, etc.)-competency-based dual strategy to improve adoptive T-cell immunotherapy to treat AML and prevent disease relapse.

## Data Availability

The original datasets are presented in the article and Supplementary Materials. Further inquiries can be directed to the corresponding author.

## Supplementary Materials

The following supporting information can be downloaded at: https://www.mdpi.com/article/10.3390/biomedicines12102250/s1, Figure S1: High glucose significantly reduced T-cell cluster formation and proliferation; Table S1: List of reagents used in this study; Table S2: List of primers (Origene, etc.) used in this study; Table S3: List of AML patients; Table S4: Increased cytokine profile from Figure 3C in GLUT1-T cells associated with metabolism or enhanced T-cell fitness compared to GFP-T cells [89,90,91,92,93,94,95,96,97,98,99,100,101,102,103,104,105,106,107,108,109,110,111,112,113,114,115,116,117,118,119,120,121,122,123,124,125,126,127,128,129,130,131,132].
