# Supplementary material for: Development of a Competitive Nutrient-Based T-Cell Immunotherapy Designed to Block the Adaptive Warburg Effect in Acute Myeloid Leukemia
Source: Biomedicines. 2024 Oct 3;12(10):2250. doi: 10.3390/biomedicines12102250 (PMC11504511; doi:10.3390/biomedicines12102250)
Supplement: Supplementary file 1 [file biomedicines-12-02250-s001.zip › biomedicines-3200320-supplementary.pdf]

Article

Development of a Competitive Nutrient-Based T-Cell Immunotherapy Designed to Block the Adaptive Warburg Effect in Acute Myeloid Leukemia

Huynh Cao [1,2,†], Jeffrey Xiao [3,†], David J. Baylink [3], Vinh Nguyen [3], Nathan Shim [3], Jae Lee [3], Dave J.R. Mallari [3], Samiksha Wasnik [3], Saied Mirshahidi [1,2,4], Chien-Shing Chen [1,2], Hisham Abdel-Azim [1,5,6], Mark E. Reeves [1,2] and Yi Xu [1,2,3,*]

[1] Division of Hematology and Oncology, Department of Medicine, School of Medicine, Loma Linda University, Loma Linda, CA 92354, USA
[2] Cancer Center, Loma Linda University, Loma Linda, CA 92354, USA
[3] Division of Regenerative Medicine, Department of Medicine, School of Medicine, Loma Linda University, Loma Linda, CA 92354, USA
[4] Biospecimen Laboratory, Department of Medicine and Basic Sciences, School of Medicine, Loma Linda University, Loma Linda, CA 92354, USA
[5] Division of Transplant and Cell Therapy, Loma Linda University Cancer Center, Loma Linda, CA 92354, USA
[6] Division of Hematology and Oncology, Department of Pediatrics, Loma Linda University, Loma Linda, CA 92354, USA
* Correspondence: dyxu@llu.edu; Tel.: +1-909-651-5887
† These authors contributed equally to this work.

Supplementary Table S1. List of Reagents used in this study.

List of Reagents				
**Antibody/Reagents**	**Color**	**Cat. #**	**Company**	**Species Reactivity**
**CD3**	PE/Cyanine7	300420	Biolegend	Human
**CD3**	FITC	300406	Biolegend	Human
**CD33**	APC	303408	Biolegend	Human
**CD33**	PE	303403	Biolegend	Human
**Ki67**	APC	350513	Biolegend	Human
**Ki67**	PE	350504	Biolegend	Human
**GLUT1**	PE	SC-377228	SantaCruz Biotech	Human
**TFAM**		MA5-16148	ThermoFisher Sci	Human
**PE anti-mouse IgG**	PE	406607	Biolegend	Mouse
**Viability Dye eFluor™ 780**		65-0865-14	eBioscience	
**PE IgG Isotype Ctrl**	PE	400111	Biolegend	Mouse
**APC IgG Isotype Ctrl**	APC	400121	Biolegend	Mouse
**CD3 MicroBeads**	FITC	130-050-101	Milteny Biotec	Human
**Dynabeads® Human T-Activator CD3/CD28**		11161D	Gibco	Human
**Proteome Profiler Human XL Cytokine Array Kit**		ARY022B	R&D	Human
**MEBCYTO Apoptosis Kit**		4700	MBL	
**2-NBDG**		HY-116215	MedChemExpres	
**IL-2**		200-02	Peprotech	Human

Supplementary Table S2. List of Primers (Origene, etc.) used in this study.

#	Name (HUMAN)	Forward Sequence	Reverse Sequence
1	***GLUT1 (SLC2A1)***	TTGCAGGCTTCTCCAACTGGAC	CAGAACCAGGAGCACAGTGAAG
2	***TFAM***	GTGGTTTTCATCTGTCTTGGCAAG	TTCCCTCCAACGCTGGGCAATT
3	***IL-2***	AGAACTCAAACCTCTGGAGGAAG	GCTGTCTCATCAGCATATTCACAC
4	***NUR77 (NR4A1)***	GGACAACGCTTCATGCCAGCAT	CCTTGTTAGCCAGGCAGATGTAC
5	***CDK1***	GGAAACCAGGAAGCCTAGCATC	GGATGATTCAGTGCCATTTTGCC
6	***CYCLINB1 (CCNB1)***	GACCTGTGTCAGGCTTTCTCTG	GGTATTTTGGTCTGACTGCTTGC

Supplementary Table S3. List of AML patients.

Sample ID	Age	Sex	Cytogenetics (Karyotype)	Gene Mutation
**AML1**	40	M	Normal	KRAs, NRAS, NPM1, DNMT3A, IDH2
**AML2**	67	M	Normal	NPM1, IDH2
**AML3**	20	M	46, XY, inv(16)(p13.1q22.1)[23]	KIT
**AML4**	63	M	46,XY,i(21)(q10)[20]	RUNX1, WT1
**AML5**	38	F	Normal	FLT3, WT1, NPM1

Supplementary Table S4. Increased Cytokine Profile from Figure 3C in GLUT1-T cells Associated with Metabolism or Enhanced T Cell Fitness Compared to GFP-T cells.

Cytokine	Coordinates	Relevant Function
Angiopoietin-2	A11, A12	Angiopoietin-2 has been shown to enhance metabolic homeostasis and improve glucose and lipid metabolism[89].
Brain-derived neurotrophic factor (BDNF)	A15, A16	Peripheral BDNF regulates the differentiation, proliferation, and survival of T lymphocytes through an anti-apoptotic effect[90]. Also, recent evidence suggests that peripheral BDNF can help sustain systemic metabolism through mitochondrial dynamic regulation[91].
CD14	A19, A20	Naïve CD4+ and CD8+ T cells proliferate, activate, and mature through interacting with CD14+ cells[92].
CD30	A21, A22	CD30 upregulates proliferation and immune response of T cells[93].
C reactive protein (CRP)	B9, B10	Elevated CRP is a sensitive marker for metabolic inflammation[94].
Epidermal Growth Factor (EGF)	B19, B20	Genetically engineered CD8+ T cells with increased EGF signaling displayed enhanced proliferation, increased production of IFN-γ and TNF-, greater therapeutic efficacy against tumors, and lower PD1, TIGIT, and LAG3 exhaustion markers[95].
CXCL5	C3, C4	CXCL5 recruits T cells to specific regions of immune response as high CXCL5 levels lead to higher T cell infiltration[96].
Fas Ligand	C7, C8	Fas ligand expression is drastically increased upon T cell activation and maintain T cell homeostasis[97].
FGF basic (FGF2)	C9, C10	FGF2 expression linked to greater CD4+ T cell infiltration[98].
FGF-7 (KGF)	C11, C12	KGF administration improved thymopoiesis and peripheral T cell numbers[99].
FGF-19	C13, C14	FGF19 increases glucose uptake and regulates glucose metabolism[100].
FLT-3 Ligand	C15, C16	FLT3 Ligand stimulates expansion of CD4+ and CD8+ naïve and memory T cells[101].
GDF-15	C19, C20	GDF15 regulates Th2 cytokines' positive effects on systemic glucose metabolism[102].
GM-CSF	C21, C22	GM-CSF drives anti-tumor responses of CD4+ and CD8+ T cells[103].
HGF	D5, D6	HGF can direct T cell homing[104].
ICAM-1	D7, D8	T cells expressing ICAM-1 can produce a co-stimulatory signal to activate T cells and program CD8 memory T cells[105].
IGFBP-3	D13, D14	IGFBP-3 regulates metabolic and mitochondrial homeostasis[106].
IL-1β	D17, D18	IL-1β expands CD4+ and CD8+ T cells that have been primed by antigens[107].
IL-3	D23, D24	IL-3 expression is nearly only present in activated T cells[108].
IL-4	E1, E2	IL-4 regulates T cell fate and is produced by activated Th2 cells[109].
IL-5	E3, E4	IL-5 induces cytotoxic T cells and is released by Th2 cells[110].

IL-6	E5, E6	IL-6 upregulates T cell proliferation and survival during inflammation[111].
IL-8	E7, E8	IL-8 is a T cell chemoattractant that is spontaneously produced by CD4+ and CD8+ T cells [112,113].
IL-10	E9, E10	IL-10 can metabolically revitalize terminally exhausted CD8+ T cells through upregulation of mitochondrial oxidative phosphorylation and increasing their proliferation and efficacy[114].
IL-12 p70	E13, E14	IL-12 improves cytotoxic efficacy of T cells and CAR T cells expressing IL-12 has an increased competitive advantage in eradicating cancer cells[115].
IL-13	E15, E16	CD4+ Th2 cells produce IL-13[116].
IL-15	E17, E18	IL-15 cytokine expression improves CAR T cells' antitumor efficacy and metabolic fitness[117].
IL-17A	E21, E22	Th17 cells secrete IL-17A and those CD4+ cells exposed to IL-17 were less capable of being suppressed[118].
IL-22	F3, F4	IL-22 is produced by T cells and as a cytokine, has been shown to drive increased oxidative phosphorylation, glycolysis, mitochondrial fitness, and metabolic reprogramming[119].
IL-34	F17, F18	IL-34 stimulates Th17 proliferation[120].
CXCL10	F19, F20	CXCL10 is a chemoattract for activated T cells and recruits CD8+ and Th1 CD4 T cells to inflamed tissues[121].
CXCL11	F21, F22	CXCL11 regulates T cell infiltration of tumors and CXCL11 expression is correlated with CD8+ T cells in the tumor microenvironment[122].
Leptin	G1, G2	Leptin is vital for glucose uptake and glucose metabolism in activated T cells that aids their survival and function[123].
CCL2	G7, G8	CCL2 is a chemoattractant for T cells and can stimulate memory T cells to produce inflammatory cytokines[124].
RANTES (CCL5)	H15, H16	RANTES is a chemoattractant for T cells and is secreted by cytotoxic T cells[125].
RBP-4	H17, H18	RBP-4 stimulates antigen presenting cells which activate CD4 T cells[126].
Resistin	H21, H22	Resistin promotes CD4+ T cell chemotaxis[127].
CXCL12	H23, H24	CXCL12 is a chemoattractant for T cells and stimulates T cell responses[128].
TFF3	I9, I10	TFF3 promotes Th17 responses and has been shown to promote the proliferation, differentiation, and anti-apoptosis of Jurkat T cells so it has been suggested to regulate T cell proliferation and survival[129].
CD71	I11, I12	CD71 is a T cell activation marker as CD71 is positively correlated with Ki67 expression in human T cells and more specifically for CD8+ T cells than CD4[130].
TNF-α	I17, I18	TNF-α is secreted by activated naïve CD4+ T cells and stimulates their differentiation into Th1 and Th17 cells and promotes metabolic reprogramming by increasing glycolysis, mitochondrial oxidation, and mitochondrial biogenesis[131]. TNF-α is also necessary for CD8+ T cell responses[132].

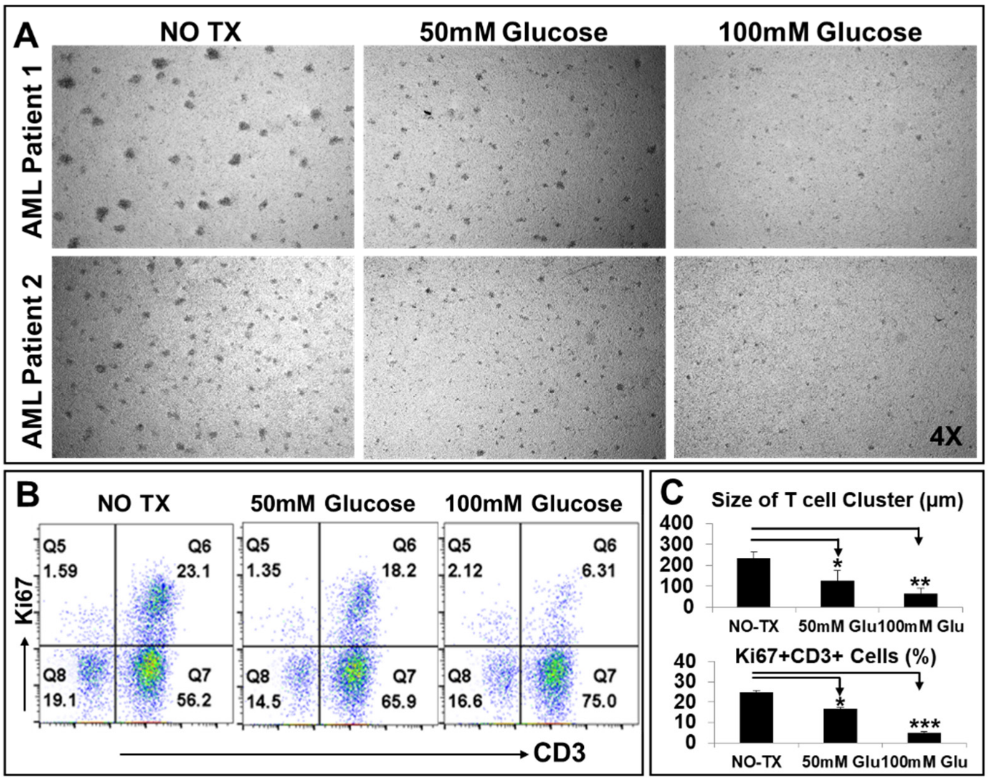

Supplementary Figure S1. High glucose significantly reduced T cell cluster formation and proliferation.

A) Representative phase-bright image (4X) of T cells isolated from AML patient peripheral blood (PB);

B) Representative flow cytometry (FC) plots show Ki67+ expression in AML PB-derived T cells which were pretreated with different doses of glucose;

C) Upper panel: Cumulative size data of T cell clusters in different experimental groups of ***A)***; Lower panel: Cumulative percentage data of Ki67+ cells in different experimental groups of ***B)***;

Where applicable, data are means ± SEM. *P<0.05, **P<0.01, ***P<0.005, n=3.

REFERENCES

89. An, Y.A.; Sun, K.; Joffin, N.; Zhang, F.; Deng, Y.; Donze, O.; Kusminski, C.M.; Scherer, P.E. Angiopoietin-2 in white adipose tissue improves metabolic homeostasis through enhanced angiogenesis. *Elife* **2017**, *6*, doi:10.7554/eLife.24071.
90. Wang, N.; Tian, B. Brain-derived neurotrophic factor in autoimmune inflammatory diseases (Review). *Exp Ther Med* **2021**, *22*, 1292, doi:10.3892/etm.2021.10727.
91. Iu, E.C.Y.; Chan, C.B. Is Brain-Derived Neurotrophic Factor a Metabolic Hormone in Peripheral Tissues? *Biology (Basel)* **2022**, *11*, doi:10.3390/biology11071063.

92. Burt, T.D.; Seu, L.; Mold, J.E.; Kappas, A.; McCune, J.M. Naive human T cells are activated and proliferate in response to the heme oxygenase-1 inhibitor tin mesoporphyrin. *J Immunol* **2010**, *185*, 5279-5288, doi:10.4049/jimmunol.0903127.
93. Cui, D.; Zhang, Y.; Chen, L.; Du, H.; Zheng, B.; Huang, M.; Li, X.; Wei, J.; Chen, Q. CD30 plays a role in T-dependent immune response and T cell proliferation. *FASEB J* **2024**, *38*, e23365, doi:10.1096/fj.202301747RR.
94. Ridker, P.M.; Buring, J.E.; Cook, N.R.; Rifai, N. C-reactive protein, the metabolic syndrome, and risk of incident cardiovascular events: an 8-year follow-up of 14 719 initially healthy American women. *Circulation* **2003**, *107*, 391-397, doi:10.1161/01.cir.0000055014.62083.05.
95. Lozano, T.; Chocarro, S.; Martin, C.; Lasarte-Cia, A.; Del Valle, C.; Gorraiz, M.; Sarrion, P.; Ruiz de Galarreta, M.; Lujambio, A.; Hervas-Stubbs, S.; et al. Genetic Modification of CD8(+) T Cells to Express EGFR: Potential Application for Adoptive T Cell Therapies. *Front Immunol* **2019**, *10*, 2990, doi:10.3389/fimmu.2019.02990.
96. Zhang, W.; Wang, H.; Sun, M.; Deng, X.; Wu, X.; Ma, Y.; Li, M.; Shuoa, S.M.; You, Q.; Miao, L. CXCL5/CXCR2 axis in tumor microenvironment as potential diagnostic biomarker and therapeutic target. *Cancer Commun (Lond)* **2020**, *40*, 69-80, doi:10.1002/cac2.12010.
97. Norian, L.A.; Latinis, K.M.; Eliason, S.L.; Lyson, K.; Yang, C.; Ratliff, T.; Koretzky, G.A. The regulation of CD95 (Fas) ligand expression in primary T cells: induction of promoter activation in CD95LP-Luc transgenic mice. *J Immunol* **2000**, *164*, 4471-4480, doi:10.4049/jimmunol.164.9.4471.
98. Chen, H.; Du, X. Increased FGF2 expression promotes immune cell infiltration and correlates with an unfavorable prognosis in thyroid cancer. *Heliyon* **2024**, *10*, e32272, doi:10.1016/j.heliyon.2024.e32272.
99. Alpdogan, O.; Hubbard, V.M.; Smith, O.M.; Patel, N.; Lu, S.; Goldberg, G.L.; Gray, D.H.; Feinman, J.; Kochman, A.A.; Eng, J.M.; et al. Keratinocyte growth factor (KGF) is required for postnatal thymic regeneration. *Blood* **2006**, *107*, 2453-2460, doi:10.1182/blood-2005-07-2831.
100. Lan, T.; Morgan, D.A.; Rahmouni, K.; Sonoda, J.; Fu, X.; Burgess, S.C.; Holland, W.L.; Kliewer, S.A.; Mangelsdorf, D.J. FGF19, FGF21, and an FGFR1/beta-Klotho-Activating Antibody Act on the Nervous System to Regulate Body Weight and Glycemia. *Cell Metab* **2017**, *26*, 709-718 e703, doi:10.1016/j.cmet.2017.09.005.
101. Parajuli, P.; Mosley, R.L.; Pisarev, V.; Chavez, J.; Ulrich, A.; Varney, M.; Singh, R.K.; Talmadge, J.E. Flt3 ligand and granulocyte-macrophage colony-stimulating factor preferentially expand and stimulate different dendritic and T-cell subsets. *Exp Hematol* **2001**, *29*, 1185-1193, doi:10.1016/s0301-472x(01)00722-6.
102. Lee, S.E.; Kang, S.G.; Choi, M.J.; Jung, S.B.; Ryu, M.J.; Chung, H.K.; Chang, J.Y.; Kim, Y.K.; Lee, J.H.; Kim, K.S.; et al. Growth Differentiation Factor 15 Mediates Systemic Glucose Regulatory Action of T-Helper Type 2 Cytokines. *Diabetes* **2017**, *66*, 2774-2788, doi:10.2337/db17-0333.
103. Kumar, A.; Taghi Khani, A.; Sanchez Ortiz, A.; Swaminathan, S. GM-CSF: A Double-Edged Sword in Cancer Immunotherapy. *Front Immunol* **2022**, *13*, 901277, doi:10.3389/fimmu.2022.901277.
104. Komarowska, I.; Coe, D.; Wang, G.; Haas, R.; Mauro, C.; Kishore, M.; Cooper, D.; Nadkarni, S.; Fu, H.; Steinbruchel, D.A.; et al. Hepatocyte Growth Factor Receptor c-Met

Instructs T Cell Cardiotropism and Promotes T Cell Migration to the Heart via Autocrine Chemokine Release. *Immunity* **2015**, *42*, 1087-1099, doi:10.1016/j.immuni.2015.05.014.
105. Bui, T.M.; Wiesolek, H.L.; Sumagin, R. ICAM-1: A master regulator of cellular responses in inflammation, injury resolution, and tumorigenesis. *J Leukoc Biol* **2020**, *108*, 787-799, doi:10.1002/JLB.2MR0220-549R.
106. Stuard, W.L.; Titone, R.; Robertson, D.M. IGFBP-3 functions as a molecular switch that mediates mitochondrial and metabolic homeostasis. *FASEB J* **2022**, *36*, e22062, doi:10.1096/fj.202100710RR.
107. Ben-Sasson, S.Z.; Wang, K.; Cohen, J.; Paul, W.E. IL-1beta strikingly enhances antigen-driven CD4 and CD8 T-cell responses. *Cold Spring Harb Symp Quant Biol* **2013**, *78*, 117-124, doi:10.1101/sqb.2013.78.021246.
108. Ryan, G.R.; Vadas, M.A.; Shannon, M.F. T-cell functional regions of the human IL-3 proximal promoter. *Mol Reprod Dev* **1994**, *39*, 200-207, doi:10.1002/mrd.1080390213.
109. Silva-Filho, J.L.; Caruso-Neves, C.; Pinheiro, A.A.S. IL-4: an important cytokine in determining the fate of T cells. *Biophys Rev* **2014**, *6*, 111-118, doi:10.1007/s12551-013-0133-z.
110. Apostolopoulos, V.; McKenzie, I.F.; Lees, C.; Matthaei, K.I.; Young, I.G. A role for IL-5 in the induction of cytotoxic T lymphocytes in vivo. *Eur J Immunol* **2000**, *30*, 1733-1739, doi:10.1002/1521-4141(200006)30:6<1733::AID-IMMU1733>3.0.CO;2-E.
111. Li, B.; Jones, L.L.; Geiger, T.L. IL-6 Promotes T Cell Proliferation and Expansion under Inflammatory Conditions in Association with Low-Level RORgammat Expression. *J Immunol* **2018**, *201*, 2934-2946, doi:10.4049/jimmunol.1800016.
112. Taub, D.D.; Anver, M.; Oppenheim, J.J.; Longo, D.L.; Murphy, W.J. T lymphocyte recruitment by interleukin-8 (IL-8). IL-8-induced degranulation of neutrophils releases potent chemoattractants for human T lymphocytes both in vitro and in vivo. *J Clin Invest* **1996**, *97*, 1931-1941, doi:10.1172/JCI118625.
113. Gesser, B.; Deleuran, B.; Lund, M.; Vestergard, C.; Lohse, N.; Deleuran, M.; Jensen, S.L.; Pedersen, S.S.; Thestrup-Pedersen, K.; Larsen, C.G. Interleukin-8 induces its own production in CD4+ T lymphocytes: a process regulated by interleukin 10. *Biochem Biophys Res Commun* **1995**, *210*, 660-669, doi:10.1006/bbrc.1995.1711.
114. Guo, Y.; Xie, Y.Q.; Gao, M.; Zhao, Y.; Franco, F.; Wenes, M.; Siddiqui, I.; Bevilacqua, A.; Wang, H.; Yang, H.; et al. Metabolic reprogramming of terminally exhausted CD8(+) T cells by IL-10 enhances anti-tumor immunity. *Nat Immunol* **2021**, *22*, 746-756, doi:10.1038/s41590-021-00940-2.
115. Kueberuwa, G.; Kalaitsidou, M.; Cheadle, E.; Hawkins, R.E.; Gilham, D.E. CD19 CAR T Cells Expressing IL-12 Eradicate Lymphoma in Fully Lymphoreplete Mice through Induction of Host Immunity. *Mol Ther Oncolytics* **2018**, *8*, 41-51, doi:10.1016/j.omto.2017.12.003.
116. Pahl, A.; Zhang, M.; Kuss, H.; Szelenyi, I.; Brune, K. Regulation of IL-13 synthesis in human lymphocytes: implications for asthma therapy. *Br J Pharmacol* **2002**, *135*, 1915-1926, doi:10.1038/sj.bjp.0704656.
117. Alizadeh, D.; Wong, R.A.; Yang, X.; Wang, D.; Pecoraro, J.R.; Kuo, C.F.; Aguilar, B.; Qi, Y.; Ann, D.K.; Starr, R.; et al. IL15 Enhances CAR-T Cell Antitumor Activity by Reducing mTORC1 Activity and Preserving Their Stem Cell Memory Phenotype. *Cancer Immunol Res* **2019**, *7*, 759-772, doi:10.1158/2326-6066.CIR-18-0466.

118. Crawford, M.P.; Sinha, S.; Renavikar, P.S.; Borcherding, N.; Karandikar, N.J. CD4 T cell-intrinsic role for the T helper 17 signature cytokine IL-17: Effector resistance to immune suppression. *Proc Natl Acad Sci U S A* **2020**, *117*, 19408-19414, doi:10.1073/pnas.2005010117.
119. Chen, W.; Zai, W.; Fan, J.; Zhang, X.; Zeng, X.; Luan, J.; Wang, Y.; Shen, Y.; Wang, Z.; Dai, S.; et al. Interleukin-22 drives a metabolic adaptive reprogramming to maintain mitochondrial fitness and treat liver injury. *Theranostics* **2020**, *10*, 5879-5894, doi:10.7150/thno.43894.
120. Li, X.; Lei, Y.; Gao, Z.; Zhang, B.; Xia, L.; Lu, J.; Shen, H. Effect of IL-34 on T helper 17 cell proliferation and IL-17 secretion by peripheral blood mononuclear cells from rheumatoid arthritis patients. *Sci Rep* **2020**, *10*, 22239, doi:10.1038/s41598-020-79312-z.
121. Peperzak, V.; Veraar, E.A.; Xiao, Y.; Babala, N.; Thiadens, K.; Brugmans, M.; Borst, J. CD8+ T cells produce the chemokine CXCL10 in response to CD27/CD70 costimulation to promote generation of the CD8+ effector T cell pool. *J Immunol* **2013**, *191*, 3025-3036, doi:10.4049/jimmunol.1202222.
122. Li, Y.; Han, S.; Wu, B.; Zhong, C.; Shi, Y.; Lv, C.; Fu, L.; Zhang, Y.; Lang, Q.; Liang, Z.; et al. CXCL11 Correlates with Immune Infiltration and Impacts Patient Immunotherapy Efficacy: A Pan-Cancer Analysis. *Front Immunol* **2022**, *13*, 951247, doi:10.3389/fimmu.2022.951247.
123. Saucillo, D.C.; Gerriets, V.A.; Sheng, J.; Rathmell, J.C.; Maciver, N.J. Leptin metabolically licenses T cells for activation to link nutrition and immunity. *J Immunol* **2014**, *192*, 136-144, doi:10.4049/jimmunol.1301158.
124. Kadomoto, S.; Izumi, K.; Mizokami, A. Roles of CCL2-CCR2 Axis in the Tumor Microenvironment. *Int J Mol Sci* **2021**, *22*, doi:10.3390/ijms22168530.
125. Appay, V.; Dunbar, P.R.; Cerundolo, V.; McMichael, A.; Czaplewski, L.; Rowland-Jones, S. RANTES activates antigen-specific cytotoxic T lymphocytes in a mitogen-like manner through cell surface aggregation. *Int Immunol* **2000**, *12*, 1173-1182, doi:10.1093/intimm/12.8.1173.
126. Moraes-Vieira, P.M.; Yore, M.M.; Dwyer, P.M.; Syed, I.; Aryal, P.; Kahn, B.B. RBP4 activates antigen-presenting cells, leading to adipose tissue inflammation and systemic insulin resistance. *Cell Metab* **2014**, *19*, 512-526, doi:10.1016/j.cmet.2014.01.018.
127. Walcher, D.; Hess, K.; Berger, R.; Aleksic, M.; Heinz, P.; Bach, H.; Durst, R.; Hausauer, A.; Hombach, V.; Marx, N. Resistin: a newly identified chemokine for human CD4-positive lymphocytes. *Cardiovasc Res* **2010**, *85*, 167-174, doi:10.1093/cvr/cvp278.
128. Smith, X.; Schneider, H.; Kohler, K.; Liu, H.; Lu, Y.; Rudd, C.E. The chemokine CXCL12 generates costimulatory signals in T cells to enhance phosphorylation and clustering of the adaptor protein SLP-76. *Sci Signal* **2013**, *6*, ra65, doi:10.1126/scisignal.2004018.
129. Lin, Z.; Zhang, J.; Duan, T.; Yang, J.; Yang, Y. Trefoil factor 3 can stimulate Th17 cell response in the development of type 2 diabetes mellitus. *Sci Rep* **2024**, *14*, 10340, doi:10.1038/s41598-024-60426-7.
130. Motamedi, M.; Xu, L.; Elahi, S. Correlation of transferrin receptor (CD71) with Ki67 expression on stimulated human and mouse T cells: The kinetics of expression of T cell activation markers. *J Immunol Methods* **2016**, *437*, 43-52, doi:10.1016/j.jim.2016.08.002.
131. Bishop, E.L.; Gudgeon, N.; Fulton-Ward, T.; Stavrou, V.; Roberts, J.; Boufersaoui, A.; Tennant, D.A.; Hewison, M.; Raza, K.; Dimeloe, S. TNF-alpha signals through ITK-Akt-

mTOR to drive CD4(+) T cell metabolic reprogramming, which is dysregulated in rheumatoid arthritis. *Sci Signal* **2024**, *17*, eadg5678, doi:10.1126/scisignal.adg5678.
132. Ye, L.L.; Wei, X.S.; Zhang, M.; Niu, Y.R.; Zhou, Q. The Significance of Tumor Necrosis Factor Receptor Type II in CD8(+) Regulatory T Cells and CD8(+) Effector T Cells. *Front Immunol* **2018**, *9*, 583, doi:10.3389/fimmu.2018.00583.
